# Parasite clearance and protection from *Plasmodium falciparum* infection (PCPI): a three-arm, parallel, double-blinded, placebo-controlled, randomised trial of presumptive sulfadoxine-pyrimethamine versus sulfadoxine-pyrimethamine plus amodiaquine versus artesunate monotherapy among asymptomatic children 3–5 years of age in Cameroon

**DOI:** 10.1186/s12879-024-09868-y

**Published:** 2024-09-26

**Authors:** Rosario Martinez-Vega, Wilfred Fon Mbacham, Innocent Ali, Akindeh Nji, Andria Mousa, Khalid B. Beshir, Ana Chopo-Pizarro, Harparkash Kaur, Lucy Okell, Helle Hansson, Emma Filtenborg Hocke, Michael Alifrangis, Roland Gosling, Cally Roper, Colin Sutherland, R. Matthew Chico

**Affiliations:** 1 Department of Disease Control, Faculty of Infectious and Tropical Diseases, London School of Hygiene & Tropical Medicine, Keppel Street, London, WC1E 7HT UK; 2 Fobang Institute for Innovation in Science and Technology, 14 Missionary Road, Simbok, Yaounde, Cameroon; 3 Department of Biochemistry, Faculty of Sciences, The University of Yaounde I, Yaounde, Cameroon; 4 Department of Biochemistry, Faculty of Science, University of Dschang, BP 67, Dschang, Cameroon; 5 The Biotechnology Center, The University of Yaounde I, Yaounde, Cameroon; 6 Department of Infection Biology, Faculty of Infectious and Tropical Diseases, London School of Hygiene & Tropical Medicine, Keppel Street, London, WC1E 7HT UK; 7 Department of Infectious Disease Epidemiology, MRC Centre for Global Infectious Disease Analysis, Imperial College London, London, W12 0BZ United Kingdom; 8 Department of Clinical Research, Faculty of Infectious and Tropical Diseases, London School of Hygiene & Tropical Medicine, Keppel Street, London, WC1E 7HT UK; 9 Department of Immunology and Microbiology, Centre for translational Medicine and Parasitology, University of Copenhagen, Copenhagen, Denmark; 10 Department of Infectious Diseases, Copenhagen University Hospital, Copenhagen, Denmark

**Keywords:** Perennial malaria chemoprevention, Intermittent preventive treatment, Antimalarial resistance

## Abstract

**Background:**

The World Health Organization 2022 malaria chemoprevention guidelines recommend providing a full course of antimalarial treatment at pre-defined intervals, regardless of malaria status to prevent illness among children resident in moderate to high perennial malaria transmission settings as perennial malaria chemoprevention (PMC) with sulfadoxine-pyrimethamine (SP). The *dhps* I431V mutation circulating in West Africa has unknown effect on SP protective efficacy.

**Methods:**

This protocol is for a three-arm, parallel, double-blinded, placebo-controlled, randomised trial in Cameroon among children randomly assigned to one of three directly-observed treatment groups: (i) Group 1 (*n* = 450) receives daily artesunate (AS) placebo on days − 7 to -1, then active SP plus placebo amodiaquine (AQ) on day 0, and placebo AQ on days 1 and 2; (ii) Group 2 (*n* = 250) receives placebo AS on days − 7 to -1, then active SP and AQ on day 0, and active AQ on days 1 and 2; and (iii) Group 3 (*n* = 200) receives active AS on days − 7 to -1, then placebo SP on day 0 and placebo AQ on days 0 to 2. On days 0, 2, 5, 7, and thereafter weekly until day 28, children provide blood for thick smear slides. Dried blood spots are collected on the same days and weekly from day 28 to day 63 for quantitative polymerase chain reaction (qPCR) and genotype analyses.

**Discussion:**

Our aim is to quantify the chemopreventive efficacy of SP, and SP plus AQ, and measure the effect of the parasite genotypes associated with SP resistance on parasite clearance and protection from infection when exposed to SP chemoprevention. We will report unblinded results including: (i) time-to-parasite clearance among SP and SP plus AQ recipients who were positive on day 0 by qPCR and followed to day 63; (ii) mean duration of SP and SP plus AQ protection against infection, and (iii) mean duration of symptom-free status among SP and SP plus AQ recipients who were parasite free on day 0 by qPCR. Our study is designed to compare the 28-day follow-up of the new WHO malaria chemoprevention efficacy study protocol with extended follow-up to day 63.

**Trial registration:**

ClinicalTrials.gov NCT06173206; 15/12/2023.

**Supplementary Information:**

The online version contains supplementary material available at 10.1186/s12879-024-09868-y.

## Background

The World Health Organization (WHO) recently updated malaria chemoprevention guidelines related to intermittent preventive treatment of malaria in infants (IPTi). Since the initial recommendation, additional data have documented the value of malaria chemoprevention provided to children aged 12 to 24 months. The name has been changed to perennial malaria chemoprevention (PMC) because the updated recommendation no longer limits the intervention specifically to infants and reflects the malaria transmission settings in which the intervention should be considered (moderate to high) [1]. Sulfadoxine-pyrimethamine (SP) has been widely used for chemoprevention in Africa, including for PMC. Evidence supporting PMC comes in part from a meta-analysis of six randomised placebo-controlled trials that showed SP conferred 30.3% (95% CI 19.8 to 39.4, *p* < 0.0001) protection against clinical malaria, 21.3% (95% CI: 8.2–32.5%, *p* = 0.002) the risk of anaemia, 38.1% (95% CI: 12.5–56.2%, *p* = 0.007) hospital admissions due to malaria, and 22.9% (95% CI: 10.0–34.0%, *p* = 0.001) all-cause hospital admissions [2–8]. A more recent meta-analysis involving nine trials showed a lower pooled effect: 22% (95% CI: 12–31%, *p* < 0.0001) against clinical malaria, 18% (95% CI: 2–32%, *p* = 0.03) against anaemia, 15% (95% CI: 7–22%, *p* = 0.0005) against hospital admission, and no significant impact against severe malaria or all-cause mortality [9]. Molecular biomarkers of SP resistance, however, vary in different malaria-endemic settings and have been shown to compromise the effectiveness of chemoprevention [10, 11].

In West Africa, specifically the Sahel region, there are emerging parasite genotypes that harbour a distinct haplotype of *dhps*, VAGKGS, which contains *dhps* I431V and *dhps* A581G mutations but lacks the *dhps* K540E [12–14]. The VAGKGS haplotype and related VAGKAS and VAGKAA have been reported in 2–40% of *Plasmodium falciparum* parasites in Cameroon, Chad, Niger and Nigeria [14]. The current distribution of parasites harbouring these genotypes is only partially described, and the effect these mutations have on parasite susceptibility to SP remains unknown. Nevertheless, it is possible that parasites expressing *dhps*-VAGKGS pose a threat to the effectiveness of PMC with SP or other SP-containing chemoprevention strategies in affected parts of the Western and Central Africa [12].

**Fig. 1:**
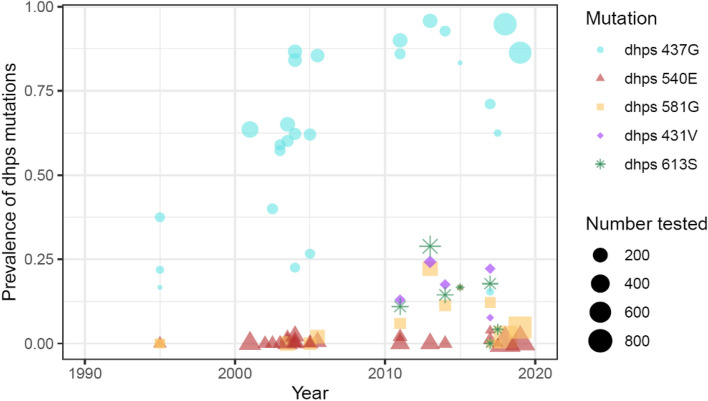
Publicly available historical data on* dhps *mutations in Cameroon. Legend: The prevalence of the *dhps* I431V genotype has risen since 2010, along with mutations in the *dhps* A581G and A613S

The *dhps* 431V variant was first identified in neighbouring Nigeria in 2009 and is now regionally widespread [12, 14–16]. In Cameroon, parasite-resistant genotypes, which include the *dhps* 431V variant, have emerged and are unevenly distributed across the country (Fig. 1), the effects of which remain unknown.

**Fig. 2:**
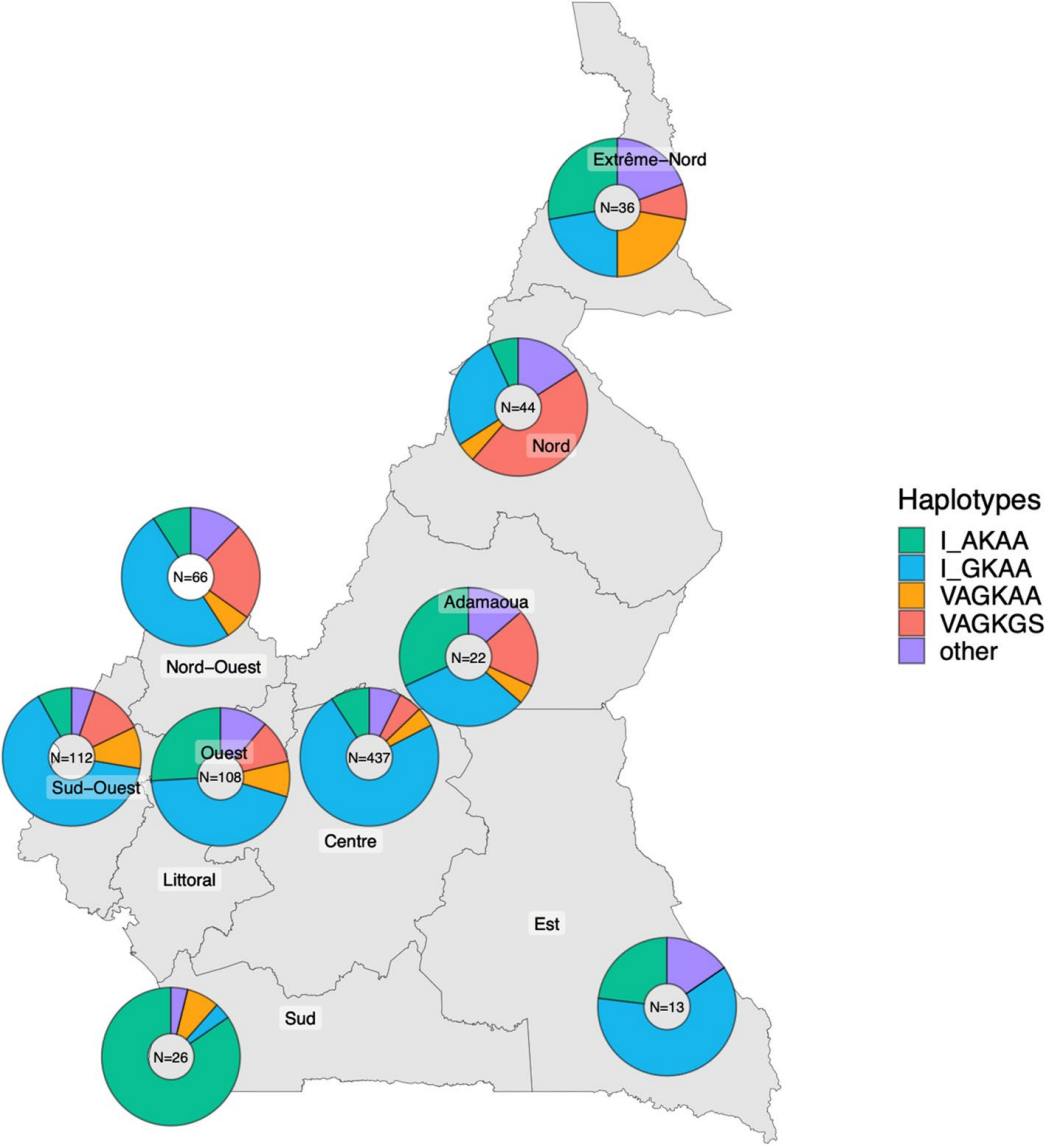
Combined published and unpublished data on frequency of *dhps* genotypes in Cameroon. Legend: The denominators shown exclude mixed infections at any of the six *dhps* loci, as well as samples with incomplete haplotype information. The red and orange sections in the pie charts show the proportion of those samples that contain the novel *dhps* 431V genotype. The question mark symbol within the haplotypes denotes a *dhps* genotype that is either 436S or 436A

There are no in vivo studies of the effect of the novel *dhps* haplotypes on SP efficacy in therapeutic studies, and no in vitro data due to the technical difficulty of estimating 50% effective concentration of sulfadoxine in conventional growth assays [17]. A high frequency of genotypes containing the *dhps* I431V mutation has been found among samples collected from the North and Extreme North regions in Cameroon, where SP plus AQ is administered for seasonal malaria chemoprevention (SMC) (Fig. 2; published and unpublished data combined). This may suggest possible selection pressure, or parasite importation from bordering areas in Nigeria where this mutation was first identified.

Our aim is to measure parasite clearance and protection from infection (PCPI) conferred by malaria chemoprevention over a 63-day period in the presence/absence of the *dhps* I431V mutation among healthy and symptom-free children between 3 and 5 years of age with unknown parasite status. Because SP may also have effects on enteric microbial communities, having been shown to increase maternal and foetal weight gain when administered as intermittent preventive treatment of malaria in pregnancy, we will also evaluate potential changes in gut microbiota associated with PMC administration [18, 19].

## Methods

### Study design

The PCPI study is a three-arm, parallel, double-blinded, placebo-controlled, randomised trial in Cameroon designed to measure the effect of various parasite genotypes associated with SP resistance on the efficacy of SP and SP plus AQ among 900 asymptomatic children between 3-5 years of age.

### Study site and recruitment

**Fig. 3:**
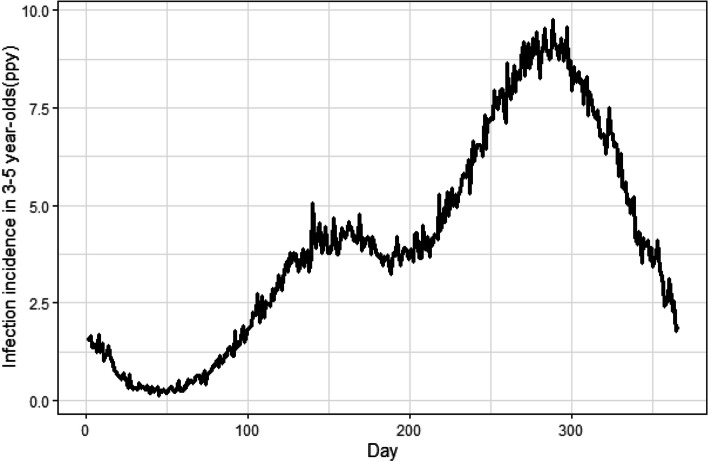
Incidence of infection per person per year across the year. Legend: Incidence was obtained by calibrating a malaria transmission model (developed by Imperial College) to a parasite prevalence 40% reported for Ngounso. The model uses historical data on intervention coverage at first administrative level (West Region) obtained from MIS and DHS surveys. The functional form for seasonality in malaria cases are based on rainfall data from the Climate Hazards Group InfraRed Precipitation with Station (CHIRPS)

The study will be conducted in the catchment area of the Ngounso and Magba health areas, situated in the Malantouen health district in the West Region of Cameroon. Malaria transmission is high over the rainy season from April to November when the prevalence among children under five years of age is approximately 40% [20] (see Fig. 3).

Staff will first conduct a community census in the study area prior to recruitment to determine the number of households and potential participants available. During household visits, study staff will provide a brief explanation of the trial and provide the family with a high-level summary of the objectives and procedures.

### Frequency of genotypes

Frequency of *dhps* 431V in Ngounso was estimated from two sets of samples collected in Ngounso and Ndop in the Northwest region in 2020 and 2018, respectively [21], as the proportion of samples containing the *dhps* 431V among those with full haplotype information and no mixed infections in any of the six dhps loci. Frequency of the *dhps* 431V mutation in Ngounso was 27.8% (5/18), similar to that estimated for Ndop (27.5%; 19/69). Further information can be found in Additional File 2.

### Eligibility criteria

The study team will recruit children who are between 3 to 5 years old and without symptoms of malaria. Parents/caregivers of the participants will provide written, informed consent before any study procedure occurs and must meet eligibility criteria.

### Inclusion criteria

To be included in the PCPI Cameroon trial, children must: (i) be 3–5 years old; (ii) exhibit no symptoms of malaria; (iii) have parents/legal guardians willing to have their child participate in all follow-up visits and seek care from study staff; and (iv) reside in the study catchment area.

### Exclusion criteria

Children are not eligible to participate in the PCPI Cameroon trial if they: (i) have evidence of acute illness as determined by clinical examination; (ii) exhibit symptoms of malaria (axillary fever ≥ 37.5 °C and / or history of fever in past 48 h); (iii) have known allergy to study medications; (iv) have received antimalarial treatment or azithromycin within 28 days prior to screening; (v) are concomitantly receiving co-trimoxazole (trimethoprim-sulfamethoxazole) for any or no indication; or (vi) are categorized as severely malnourished according to WHO child growth standards.

### Group allocation and masking

Children who meet inclusion criteria will be randomly assigned to one of three treatment groups in a ratio of 2:1:1 and based on a random block length of 6, 12, or 18. Before the onset of the study, a set of sequentially numbered, opaque sealed envelopes containing the allocation group will be prepared and stored at the district hospital. An independent statistician at the London School of Hygiene & Tropical Medicine (LSHTM) and a lead pharmacist in Cameroon, neither of whom will otherwise be involved in the study, will be responsible for treatment allocation and the preparation of study drugs.

### Sample size calculation

We used a simulation approach to calculate the statistical power and sample size needed to detect a significant difference in the duration of protection against a new infection that is either *dhps* 431I (wild-type) or dhps 431V (mutant-type). For a given sample size and assumptions on the setting epidemiology and study design, we simulated 1000 datasets and then fitted a Bayesian model to each simulated dataset. Power was defined as the proportion of simulations that rejected the null hypothesis (i.e. no significant difference in the duration of SP protection between sensitive and resistant *P. falciparum* strains). Based on malaria surveillance data from Ngounso, we assumed a *P. falciparum* slide prevalence of 40%, equating to an average malaria incidence of six infections per person per year during the transmission season, and an expected loss to follow-up rate of 10% [22]. Under this scenario, a sample size of 450 is needed in Group 1 the SP arm, 250 in Group 2 the SP plus AQ arm, and 200 in the artesunate (AS) arm, Group 3 the control will have 81% power to detect a significant difference in the mean duration of SP protection against *dhps* 431 V and *dhps* 431I. A sample size of 250 was chosen for the SP plus AQ group, which provided a sufficient power to detect a difference of 35 days in the mean duration of protection against any parasite compared to AS, or a difference of 11 days compared to SP (power > 80%). The model assumes that mutation frequency at the parasite population level is the same as mutation prevalence of the sample in the given patient population among samples with unmixed infections at the codon of interest. Details on this modelling approach are detailed elsewhere [23], and an expanded description of this method applied to the PCPI study in Cameroon can be found in Additional File 2.

### Description of the intervention

Children will receive treatment according to their group assignment. After randomisation, Groups 1–2 will receive a 7-day course of placebo AS monotherapy; Group 3 will be given a 7-day course of active AS (Table 1). This will span days − 7, -6, -5, -4, -3, -2, and − 1. Then, on day 0, children in Groups 1–2 will receive a single course of malaria chemoprevention, either active SP plus AQ placebo or active SP plus active AQ; Group 3, having completed a 7-day course of AS will receive placebo SP and placebo AQ. Group 3 will reflect a cohort of children who are parasite-free at day 0 and allow for an accurate estimate of background incidence (true transmission intensity) to which all groups will be exposed during follow-up. In addition, this group allows a more accurate estimation of underlying frequency of *dhps* 431V mutations in the parasite population. We will look at parasite clearance among those who were quantitative polymerase chain reaction (qPCR)-positive at day 0 and time to incident infection among qPCR-negative at day 0.

**Table 1:** Summary of treatments and children per group

Group	Treatment	No. Children
1	**sulfadoxine-pyrimethamine (SP)** Day − 7: daily placebo artesunate monotherapy (AS) until Day 0 Day 0: sulfadoxine-pyrimethamine (SP) plus placebo amodiaquine	450
2	**sulfadoxine-pyrimethamine plus amodiaquine (SP + AQ)** Day − 7: daily placebo artesunate monotherapy (AS) until Day 0 Day 0: sulfadoxine-pyrimethamine plus amodiaquine (SP + AQ)	250
3	**artesunate monotherapy (AS)** Day − 7: daily artesunate monotherapy (AS) until Day 0 Day 0: placebo SP plus placebo amodiaquine	200

SP consists of one tablet containing 250 mg of sulfadoxine and 12,5 mg of pyrimethamine (MA158 trade name, WHO prequalified product, Macleods Pharmaceuticals Ltd, Mumbai, India) given as a single oral dose for 1 day. SP plus AQ consists of two tablets containing 500 mg of sulfadoxine and 25 mg of pyrimethamine, and 153 mg of amodiaquine (as hydrochloride) (MA117 trade name, WHO approved products, Guilin Pharmaceuticals Co., Ltd, Guilin, China), given orally once a day for one and three consecutive days, respectively. The placebo tablets will be provided by Macleods Pharmaceuticals and have same appearance as active SP and AQ, respectively. AS consists of one tablet containing 50 mg of artesunate given orally once daily for 7 consecutive days (MA044 trade name, WHO approved product, Guilin Pharmaceuticals Co., Ltd, Guilin, China). The placebo tablets will also be provided by Guilin Pharmaceuticals and have the same appearance as active AS. Information regarding the use of these medicines can be found in the patient information leaflets in Additional File 3.

### Participant follow-up

All children in Groups 1–3 will be followed 71 days total, comprising 7 days prior to day 0, defined as the first day that the two chemo-prevention groups receive their SP or SP plus AQ, day 0 itself and 63 days (9-week period total) following day 0 (see activity timeline in Table 2). The study medications will be administered as direct observed therapy by PCPI clinical staff on all days of dosing and the child will be observed for 30 min before the study team departs to ensure the medication has been properly ingested and record any adverse reactions. Daily follow-up dosing visits will be held on days − 6, -5, -4, -3, -2, -1 for all participants. Similar monitoring of adverse events (AEs) will be carried out on day 0 after dosing, and thereafter during scheduled visits on days 2, 5, 7, 14, 21, 28, 35, 42, 49, 56 and 63, and any unscheduled visit.

**Table 2:**
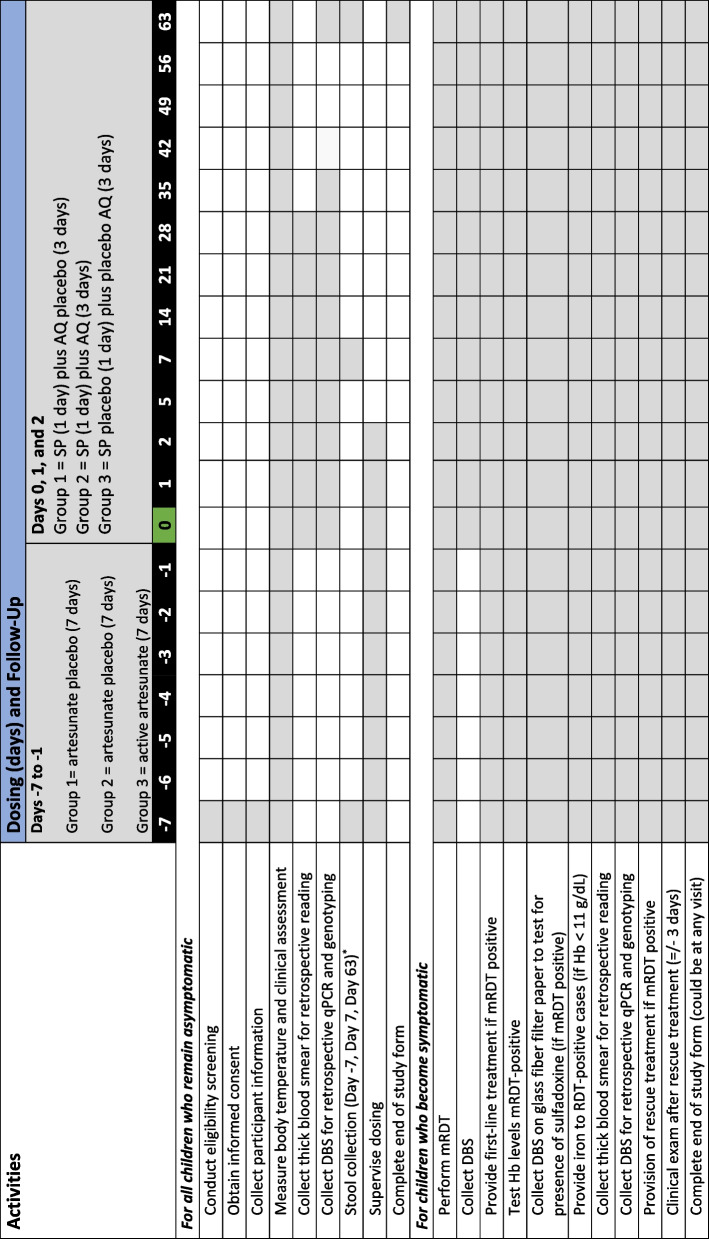
Summary of activities and follow-up period by treatment group (days)

*mRDT *Malaria rapid diagnostic test, *DBS *Dried blood spots, *qPCR *Quantitative polymerase chain reaction, *Hb *Haemoglobin

(1) Microscopy slides up to Day 28 will be collected and read for the purpose of alignment with the WHO chemoprevention protocol; (2) The timing of activities for children who become symptomatic will be based on if/when symptoms occur and, therefore, all boxes are shaded to reflect readiness to respond at any time point, or indeed on any day of symptomatic presentation. Following the provision of rescue treatment, these children will be censored and no longer contribute to trial endpoints; (3) FINISTECH staff will oversee the census work pre-initiation, sample collection and drug administration. Actual sample collection and drug administration will be carried out by community health workers/health workers from local Ministry of Health facilities and supported by the core FINISTECH investigators

Thick blood smears will be prepared from pin-prick samples collected at day 0 (pre-dose), 2, 5, 7, 14, 21, 28. Any child who is symptomatic at a scheduled or unscheduled visit from day − 7 onward will provide a pin-prick of blood for a malaria rapid diagnostic test (RDT) (SD Biosensor, Healthcare Pvt. Ltd., Gurugram, India). From Day 0 onwards any child who is symptomatic at a scheduled or unscheduled visit will provide a pin-prick of blood for a blood film, and a dried blood spot (DBS) (Whatman 3MM CHR, Cytiva, Cardiff, UK). If RDT-positive, a DBS will be collected on fibre glass filter paper (Fisherbrand, Fisher Scientific, Loughborough, UK) to test for presence of sulfadoxine. The child will be provided a full course of the first-line antimalarial treatment as recommended by the national malaria case management guidelines [24] and no longer sought for further blood collection in the study, unless the child again becomes febrile prior to the end of day 63, in which case the child will be tested by RDT again and treated if positive. Any child suspected of developing a case of severe malaria will be referred promptly to the district-level reference facility and be given treatment according to national guidelines [24]. On any occasion that a child is found to be RDT-positive, the participant will have haemoglobin (Hb) levels checked. Any child with Hb levels < 11 g/dL will receive iron supplements according to national guidelines [24]. Follow-up activities are shown in Table 2.

Study completion at the individual level will be considered achieved when a child has either reached day 63 in the follow-up period or, at any time prior, if a symptomatic child is confirmed RDT-positive and antimalarial rescue treatment has been provided. At study level, clinical trial termination will be considered when the sample size has been achieved and all participants have completed their last visit.

### Strategies for retention

As part of pre-screening procedures, parents/legal guardians will be counselled on the importance of following the study visit calendar. They will also be assessed for the probability of travelling out of the catchment area of the study prior to delivery and, therefore, being at risk of not completing the trial. If the study team believes there is a risk, the child will not be included in the study.

All parents/legal guardians will be reimbursed for compensation of time for each household visit. If the child is referred during the scheduled household visits or need to seek medical care outside these (unscheduled visits), the cost of transportation to and from the health facility will be reimbursed.

If the parents/legal guardians are not at home or available during scheduled follow-up visits, a PCPI team member will call them within 24 h to find a time when they can meet. In any case, the team member will keep a schedule for each enrolled child and a call will be provided the day prior to the day of follow-up. Children will be categorised as lost to follow-up if the parent/legal guardian repeatedly fails to be at home for more than three scheduled visits and is unable to be contacted by the study team.

Follow-up visits from day − 7 to day 5 will have a 1-day window (+/-) that will be acceptable for the corresponding scheduled visit. From day 7 onward, a 3-day window (+/-) will be acceptable for the corresponding weekly visits. Visits outside of a 3-day window over the nine-week period will be recorded as an unscheduled visit and all procedures will be followed in the manner as a scheduled visit. A participant may miss some scheduled visits and remain in the study at the discretion of the investigators, although the participant will be considered lost to follow-up and no longer in the study seven days following the final scheduled visit, i.e. day 71, if no contact occurs during this period. All attempts made to contact a participant will be documented in the medical notes.

### Participant withdrawal

Every reasonable effort will be made to maintain protocol compliance and participation in the study. A withdrawal from the investigational product is defined as any participant who does not receive the complete treatment. A parent/legal guardian may withdraw their child from the study at any time for any reason as consent is entirely voluntary. A participant may also be withdrawn at any time at the discretion of the investigator for safety, behavioural, compliance, or administrative reasons.

Parents/legal guardians may decide to withdraw their child from the study at any time by simply informing a staff member of their desire to do so. Staff will notify the Principal Investigator who, in turn, will notify the Co-Chief Investigators. If the parents/guardians want to withdraw consent for use of data and/or samples already collected, and/or to withdraw consent for long term storage and future use of data and samples, the data and samples for their child will be removed and destroyed within one month of request. If withdrawal is the result of a Serious Adverse Event (SAE), the investigator will offer to arrange for appropriate management of the event and its cost. If parent/legal guardian withdraws a child prematurely for any reason, the child will not be re-entered into the trial. The subject ID number and treatment number will not be reused. An end-of-study form will be completed at the day 63 visit and for withdrawn participants prior to the last scheduled study visit. If consent is withdrawn by the parent/legal guardian for any reason, then the last visit prior to withdrawal of consent will be considered the final visit and documented accordingly. All study withdrawals will be recorded in the participant’s medical records and in the Case Report Form.

### Adverse events

Most AEs and adverse drug reactions that occur in this study, whether they are serious or not, will be expected treatment-related side effects due to the drugs used in this study. The assignment of causality as unrelated, unlikely, possible, probable, definitely, or not assessable event will be made by the investigator responsible for the care of the participant. A data safety and monitoring board will be stablished for SAEs monitoring purposes (Additional File 4).

#### Outcome measures

The aim of the PCPI study is to measure the chemopreventive efficacy of SP, and SP plus AQ, and quantify the effect on both parasite clearance and protection from infection of the parasite genotypes associated with SP resistance in subjects receiving SP chemoprevention. Primary and secondary endpoints of interest can be found in Tables 3 and 4 respectively.

**Table 3:** Study primary endpoints

1. **Parasite clearance**
Time to clearance of parasite genotypes among SP recipients who were positive on Day 0 by qPCR and measured to Day 63. Differences will be compared by the presence or absence of *dhps* I431V
2. **Protection from infection (a)**
Mean duration of SP protection against parasite genotypes determined by *dhps* gene sequence (presence/absence of *dhps* I431V) among SP recipients who were parasite-free on Day 0 by qPCR
3. **Protection from infection (b)**
Mean duration of symptom-free status among SP recipients who were parasite free on Day 0 by qPCR, compared by *dhps* 431V genotype at time of febrile malaria episode

**Table 4:** Study secondary endpoints

1.** Parasite clearance**	**SP + AQ**	1. Time to clearance of parasite genotypes among SP + AQ recipients positive at Day 0 by qPCR and measured to Day 63. Differences will be compared by the presence or absence of *dhps* I431V
2.** Protection from infection (a)**	**SP + AQ**	1. Mean duration of SP + AQ protection by qPCR
3.** Protection from infection (b)**	**SP + AQ**	1. Mean duration of symptom-free status among SP + AQ recipients
4.** Therapeutic efficacy outcomes**	**SP**	1. Acute clinical and parasitological response (ACPR) at Day 28 by presence/absence of *dhps* I431V
		2. ACPR at Day 28 by presence/absence of *dhps* I431V + *dhps* A581G
	**SP + AQ**	3. ACPR at Day 28

#### Data collection procedures

Data will be collected using REDCap (Research Electronic Data Capture), a Good Clinical Practice (GCP)-compliant web-based application for building and managing online research surveys and databases. Only the Cameroonian investigators, Co-Chief Investigators, and the data managers will have access to this database. All data collected using REDCap will be transmitted at the end of each day and backed up at LSHTM.

At the end of the study, all documents with names or addresses will be destroyed. Data from the web-based REDCap will be used by the investigators for analysis and preparation of reports for an independent data safety monitoring board. Access is controlled by a firewall policy based on the host address and application. Activity on LSHTM servers is fully audited recording both login details and file system access. Access will be limited to essential research personnel.

At enrolment, PCPI team members will administer a household and caregiver questionnaire integrated in the REDCap. Questionnaire domains include sociodemographic and socioeconomic data. Follow-up data will be collected using REDCap according to the schedule outlined in Table 2.

#### Data analysis and laboratory methods

The deterministic version of the stochastic model used for power calculations (using the probabilistic programming language Stan in R version 4.2.2) will be used to estimate: (i) the mean duration of protection against each genotype *dhps* 431I and *dhps* 431V (and difference between the two), (ii) the protective efficacy provided by SP against each genotype (over time following treatment), (iii) the background incidence of malaria, and (iv) the underlying frequency of mutant strain (*dhps* 431V). By disaggregating the impact of these factors, protective efficacy of each drug can be estimated and applied to different settings with varying levels of transmission and resistance. Details of the model structure can be found elsewhere [23, 25].

Malaria microscopy slides will be prepared for samples collected on days 0, 2, 5, 7, 14, 21, 28 for retrospective analysis only, but will be read for children who present with symptoms and are RDT-positive. The procedures for slide preparation and reading will be detailed in the operations manual/laboratory Standard Operating Procedures (SOP). Two independent readers will record their *Plasmodium species* and parasite counts. If results are discrepant, a third tie-breaking reader will determine the final reported observation using methods previously described [26].

DBS will be collected on filter papers with a pin-prick of blood at the same time-points for parasite detection using qPCR to carry out genotyping to determine the prevalence of mutations related to resistance to SP and other drugs, and to test for the presence of sulfadoxine in subject plasma. DBS will be prepared, desiccated and securely stored under ambient temperature as described in the manual/laboratory SOPs. The use of microscopy and qPCR will allow distinguishing between recrudescent and new infections on paired samples.

Malaria parasite counting will be done as per WHO protocol as well as using a duplex qPCR employing hydrolysis probes to human and *P. falciparum* amplicons to quantify parasite density in DNA extracted from blood spots as described elsewhere [27, 28]. The proportion of patients with parasitaemia will be presented, as well as the proportion of patients with parasites containing the *dhps* mutations of interest (in Cameroon these are *dhps* I431V with or without *dhps* A581G). For positive samples, *dhps* and *dhfr* genotypes will be determined using a high-throughput pipeline established using next-generation targeted sequencing technology [29].

Stool samples will be collected and stored from a subset of 150 consenting children (50 selected participants per study arm) at three time points on days − 7, 7, and 63, as shown in the study schedule (Table 2). The intestinal microbiomes will be analysed on an exploratory basis to better understand potential changes in the gut, induced by study medication, that enhances nutrient absorption.

#### Ethical considerations and results dissemination

The investigators will ensure that this study is conducted according to the protocol and SOPs that meet the principles of the current, 7th revision of the Declaration of Helsinki 2013. 12.2. International Conference on Harmonisation guidelines for GCP in clinical studies. The study protocol and any subsequent amendments have been approved by the following four institutions before participants are enrolled: the LSHTM Ethics Committee (UK); the National Ethics Committee for Human Health Research and, separately, the Ministry of Public Health / Directorate of Pharmacy, Medicines and Laboratories (Cameroon); and the WHO Ethics Committee (Switzerland).

Results from this study will be shared with the National Malaria Control Programme, non-governmental organizations partners, and community stakeholders at the end of the study and prior to publication and dissemination.

## Discussion

Currently there are no guidelines to inform the design and analysis of trials that aim to estimate the impact of resistant markers on chemoprevention efficacy. The updated WHO chemoprevention guidelines are less prescriptive about the total number and frequency of doses, and no longer stipulate that the frequency of the *dhps* K540E must be below 50%. This specific guideline had previously discouraged countries from adopting IPTi with SP. However, this was not well supported by empirical evidence as there remains an important evidence gap concerning implementation of PMC in geographic areas with different SP resistance profiles.

This PCPI protocol will focus on the VAGKGS /VAGKAA of *dhps* genotypes which have emerged and spread in Cameroon in recent years, and for which the impact on parasite clearance and protective efficacy remains unknown. The investigators aim to quantify the protection provided by single-dose chemoprevention against different *dhps* variants, accounting for underlying frequencies of these variants in the parasite population, and background transmission intensity. This will allow extrapolation of protective efficacy to settings of different frequencies and transmission intensities, and consequently the quantification of the impact of different PMC schedules on malaria burden. The duration of follow-up in the PCPI protocol was carefully considered. Most study designs of malaria treatment efficacy have a primary endpoint of parasitaemia (present/absent) measured by slide microscopy at day 28 (4 weeks) with polymerase chain reaction (PCR) correction, an approach also outlined in the new WHO chemoprevention efficacy study (CPES) protocol [30]. Our PCPI study protocol is designed to be consistent and aligned with the WHO CPES protocol. Consequently, data derived from PCPI studies will be comparable with data from other studies, including those conducted using the CPES protocol. The CPES protocol, which is designed for routine periodic surveillance of chemoprevention implementation, defines chemoprevention efficacy as both the ability to clear existing parasites and prevent a new infection for a short period (of 28 days). In contrast, this PCPI protocol has been designed as a bespoke research tool to provide a one-off estimate of genotype-specific PMC-SP efficacy, and will separate resistance effects on these two outcomes of clearance and protection and extend the follow-up period to day 63 (9 weeks). This allows for better quantification of the protective efficacy against new infections by genotype, particularly when the mean duration of protection against more sensitive strains is higher or close to 28 days [23]. Additionally, the choice of a 63-day follow-up simulates what might be the protective efficacy in a scenario where chemoprevention is administered to children every two months. However, this also greatly adds to cost, precluding its utility for routine periodic surveillance by malaria control programmes. The PCPI approach will be valuable for comparative research through meta-analyses as other longer-acting interventions, including introduction of monoclonal therapies and malaria vaccines, becomes available. The PCPI protocol will also generate new knowledge around the potential use of alternative chemoprevention therapies including SP plus amodiaquine (SP plus AQ), the treatment currently recommended by the WHO for SMC.

### Trial status

Recruitment began on 10^th^ June 2024.

## Supplementary Information

Additional file 1. PCPI Cameroon_SPIRIT_Fillable_checklist.

Additional file 2. PCPI Cameroon_ Sample size.

Additional file 3. PCPI Cameroon_ Medicines information leaflets.

Additional file 4. Data Safety Monitoring Board Charter.

Additional file 5. Information sheets and consent forms.

## Data Availability

Anonymized data and collected filter paper dried blood spots will be available for future research that can benefit the communities, where appropriate ethical approvals are in place, and stored in institutional data repositories.

## Abbreviations

AE: Adverse event
AQ: Amodiaquine
AS: Artesunate monotherapy
CPES: Chemoprevention efficacy study protocol
DBS: Dried blood spot
*dhfr*: Dihydrofolate reductase
*dhps*: Dihydropteroate synthase
GCP: Good Clinical Practice
Hb: Haemoglobin
Ippy: Infections per person-year
IPTi: Intermittent preventive treatment of malaria in infants
LSHTM: London School of Hygiene & Tropical Medicine
mRDT: Malaria rapid diagnostic test
PCPI: Parasite clearance and protection from infection
PCR: Polymerase chain reaction
PMC: Perennial malaria chemoprevention
qPCR: Quantitative polymerase chain reaction
REDCap: Research Electronic Data Capture
SAE: Serious Adverse Event
SMC: Seasonal malaria chemoprevention
SOPs: Standard Operating Procedures
SP: Sulfadoxine-pyrimethamine
SP plus AQ: Sulfadoxine-pyrimethamine plus amodiaquine
WHO: World Health Organization
