# Supplementary material for: Parasite clearance and protection from Plasmodium falciparum infection (PCPI): a three-arm, parallel, double-blinded, placebo-controlled, randomised trial of presumptive sulfadoxine-pyrimethamine versus sulfadoxine-pyrimethamine plus amodiaquine versus artesunate monotherapy among asymptomatic children 3–5 years of age in Cameroon
Source: BMC Infect Dis. 2024 Sep 26;24:1028. doi: 10.1186/s12879-024-09868-y (PMC11425934; doi:10.1186/s12879-024-09868-y)
Supplement: Supplementary file 2 — Additional file 2. PCPI Cameroon_ Sample size. [file 12879_2024_9868_MOESM2_ESM.pdf]

Additional File 2: Sample size

Cameroon PCPI Study

Investigators and institutions involved:

London School of Hygiene and Tropical Medicine (LSHTM) – London, UK

Fobang Institute for Innovation in Science and Technology (FINISTEC) – Yaounde, Cameroon

University of Copenhagen – Copenhagen, Denmark

Imperial College London (ICL) – London, UK

Funder: UNITAID

To estimate the power provided with different sample sizes and varying scenarios we simulated the trajectories of a cohort of children being enrolled into the trial. We used stochastic models to generate these estimates based on the probability of a study participant being exposed to a *Plasmodium falciparum* (*Pf)* parasite, the probability that parasite is either 'resistant' (*dhps* 431V) or 'sensitive' (*dhps* 431I), and the probability that exposure to each parasite genotype results in a successful infection (i.e., not protected by the drug). We generated 1000 simulations, fitted a deterministic version of the model to each simulated dataset, and estimated the mean difference in protection among sensitive and resistant strains (along with associated 95% Credible Intervals). The statistical power was estimated as the percentage of simulations that rejected the null hypothesis (i.e., no difference in the mean duration of protection between the two strains). An expanded description of this method can be found in Mousa et al. [1].

Based on malaria surveillance data from Ngounso, we assumed a *Pf* slide prevalence of 40%, equating to an average malaria incidence of 6 infections per person per year during the transmission season, and a loss to follow-up of 10% based on previous studies [2]. Only children who are slide-negative on Day 0 will contribute to estimating the duration of SP protection against a new infection. Our first data point comes from a sample obtained in

Ngounso in 2020 as part study looking at drug resistance markers in pregnant women attending antenatal care where 37.5% (15/40) of genotyped samples contained the *dhps 431V* mutation. A different sample of dried blood spots collected in 2018 from Ndop in the Northwest region, located close to Ngounso, was collected as part of the Malaria Research Capacity Development in West and Central Africa (MARCAD) project looking at resistance markers in relation to SMC. Analysis of these samples suggested a *dhps* 431V prevalence of 42.3% (47/111) (unpublished data). Frequency of *dhps* 431V was estimated for both sets of samples as the proportion of samples containing the *dhps* 431V among those with full haplotype information and no mixed infections in any of the six *dhps* loci. Frequency of the *dhps* 431V mutation in Ngounso was 27.8% (5/18), similar to that estimated for Ndop (27.5%; 19/69).

In all simulations we assumed that the *dhps* 431 genotype is determined for 85% of infections, based on an analysis of samples collected during the ASPIRE trial in Zambia [3]. The infection rate, is dependent on the entomological inoculation rate (EIR) and the probability of an infectious bite leading to a successful infection. In Ngounso, given a parasite prevalence by microscopy of 40%, we expect an infection rate of approximately 6 infections per person-year (ippy) during the transmission season. This infection rate was derived from calibrating a published model of malaria transmission developed by Imperial College London to match the slide prevalence measured for Ngounso [4, 5]. Power calculations are based on methods outlined by Arnold *et al.* [6] and assumed the following conditions during the trial: (i) a duration of follow-up of 63 days, (ii) an incidence of infection of 6 infections per person per year (ippy), (iii) a frequency of resistant parasite (F_R) of 0.3, and (iv) an expected duration of SP prophylaxis of 15 days against parasites with *dhps* 431V mutations and 28 days against parasites with the wildtype *dhps* 431I (equating to an expected difference of 13 days). Due to the lack of in vivo studies, the impact of the novel *dhps* 431V mutation on SP protective efficacy is unknown. The expected durations of protection are based on estimates of duration of protection against the

sextuple and quadruple genotypes from previous studies[40-43] (an estimated difference of 20 days in protection). No prophylactic effect is expected or modelled in the AS-treated participants.

For the modelled scenario, each simulated dataset contained the number of individuals at risk, and those infected with a sensitive or resistant parasite for each of the observation time points (Days 0, 2, 5, 7, 14, 21, 28, 35, 42, 49, 56, and 63). The distribution of the proportion infected with each of the parasite strains across the simulations is shown in Figure 1 below.

Fig. 1 Distribution of the proportion of new infections with the resistant and sensitive parasite

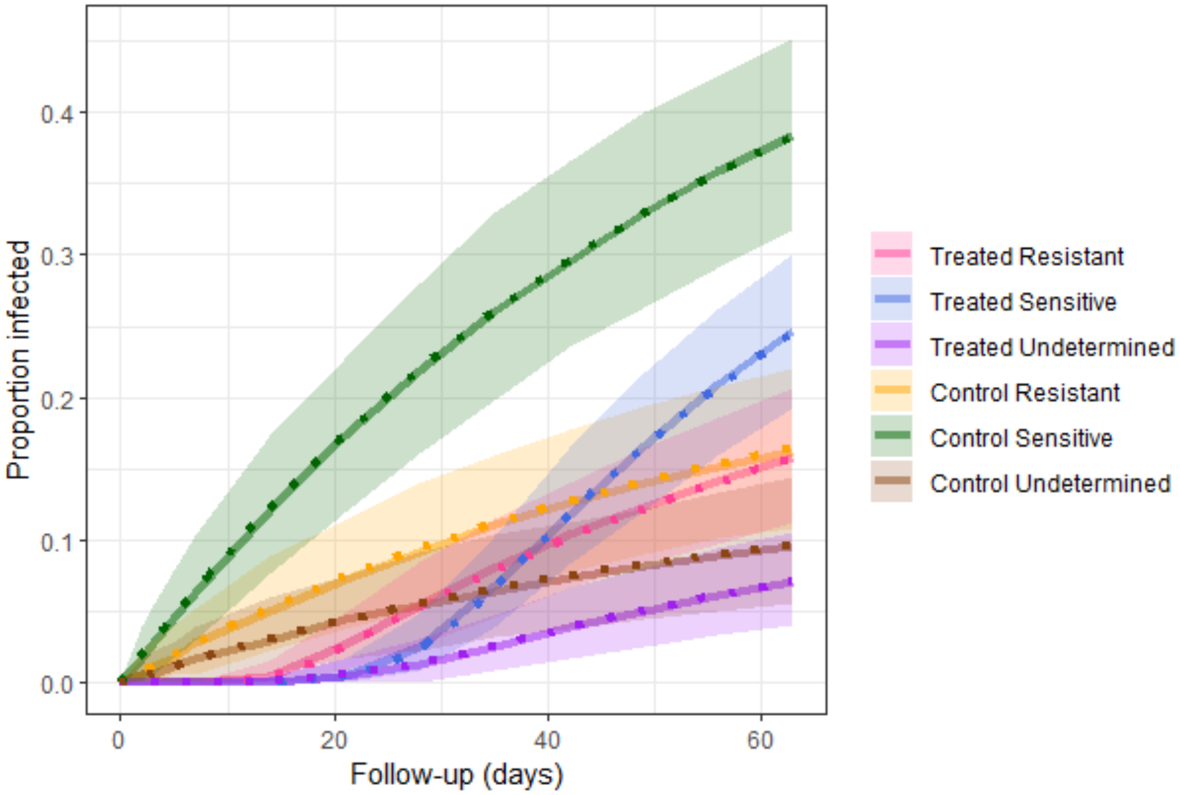

The assumptions are the following: N(SP, referred to as "treated")=450, N(AS referred to as "control")=200, 6 infections per person per year, 40% prevalence by microscopy, 10% loss to follow-up, frequency of Pfdhps I431V= 30%, 28 day protection against sensitive and 15 days protection against resistant strains, 85% probability of determining dhps 431.The solid lines

denote the median, and shaded areas show the 2.5th and 97.5th percentiles. The dotted line shows the values predicted by the deterministic model, based on the parameter inputs. The median across the 1000 simulations closely follows the deterministic values.

The deterministic version of the model was fitted to each of these simulated datasets. We used relatively uninformative priors for all parameters and ran the model each time with 5000 iterations and 4 chains (and 2000 burn-in iterations). Based on the estimated protection parameters (λ_R, λ_S, w_R, w_S) from each of these models, we estimated 1) protective efficacy following chemoprevention with SP (Figure 2A), and 2) the difference in mean duration of protection against resistant and sensitive strains (Figure 2B). A sample size of 450 in the SP arm, 200 in the AS control group and 250 in the SPAQ arm would achieve a power of 81% in estimating a significant difference in the duration of SP protection against *dhps* 431V and *dhps* 431I.

Fig. 2A Predicted protective efficacy over time against resistant dhps 431V and sensitive dhps 431I parasites

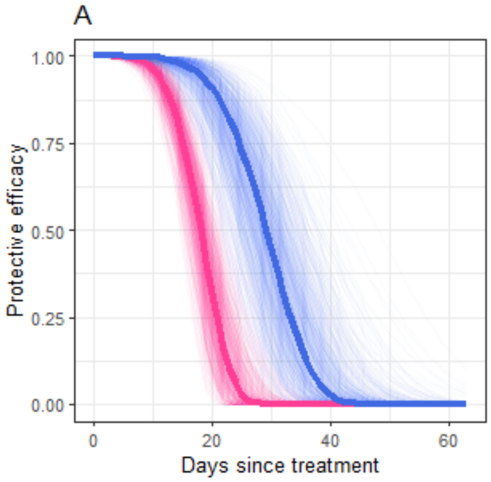

Resistant dhps 431V parasites (in pink) and sensitive dhps 431I parasites (in blue), from time since treatment. Solid line denotes the median of the medians estimated across 1000 simulations, and faint lines show all medians estimated from 1000 simulations. The plot assumes the assumptions under the baseline scenario.

Figure 2B Median posterior value for the mean duration of protection for resistant and sensitive strains

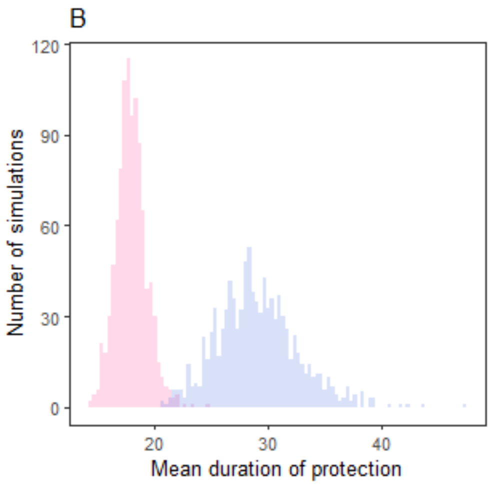

The plot assumes the assumptions under the baseline scenario, across 1000 simulations.

For treatment Group 2, the aim is to explore SPAQ as an alternative chemoprevention treatment and measure the expected duration of protection provided. For this group, genotype differences in protection will be explored but not explicitly modelled due to the limited sample size. Rather, an overall duration of protection will be estimated, and compared with AS. The duration of protection given by SP+AQ was shown to range between 28 to 35 days, based on fitting the model to reinfections following SP+AQ in a therapeutic trial in Malawi [7], and previous studies [8, 9]. Power calculations for the SP+AQ arm compared to the AS group are based on 1000 simulations per group and the p value of the log likelihood test from a Cox proportional hazards regression. A sample size of 250 was chosen for the SP+AQ group, which provided a sufficient power to detect a difference of 35 days in the mean duration of protection against *any* parasite compared to AS, or a difference of 11 days compared to SP (power>80%).

References

1. Mousa A, Cuomo-Dannenburg G, Thompson HA, Chico RM, Beshir KB, Sutherland CJ, Schellenberg D, Gosling R, Alifrangis M, Hocke EF *et al*: **Measuring protective efficacy and quantifying the impact of drug resistance: A novel malaria chemoprevention trial design and methodology**. *PLoS Med* 2024, **21**(5):e1004376.
2. Zani B, Gathu M, Donegan S, Olliaro PL, Sinclair D: **Dihydroartemisinin-piperaquine for treating uncomplicated Plasmodium falciparum malaria**. *Cochrane Database Syst Rev* 2014, **2014**(1):CD010927.
3. **The ASPIRE Trial - Aiming for Safe Pregnancies by Reducing Malaria and Infections of the Reproductive Tract** [https://clinicaltrials.gov/study/NCT04189744]
4. Griffin JT, Ferguson NM, Ghani AC: **Estimates of the changing age-burden of Plasmodium falciparum malaria disease in sub-Saharan Africa**. *Nat Commun* 2014, **5**:3136.
5. Griffin JT, Hollingsworth TD, Okell LC, Churcher TS, White M, Hinsley W, Bousema T, Drakeley CJ, Ferguson NM, Basanez MG *et al*: **Reducing Plasmodium falciparum malaria transmission in Africa: a model-based evaluation of intervention strategies**. *PLoS Med* 2010, **7**(8).
6. Arnold BF, Hogan DR, Colford JM, Hubbard AE: **Simulation methods to estimate design power: an overview for applied research**. *BMC Medical Research Methodology* 2011, **11**(1):94.
7. Grobusch MP, Lell B, Schwarz NG, Gabor J, Dornemann J, Potschke M, Oyakhirome S, Kiessling GC, Necek M, Langin MU *et al*: **Intermittent preventive treatment against malaria in infants in Gabon--a randomized, double-blind, placebo-controlled trial**. *J Infect Dis* 2007, **196**(11):1595-1602.
8. Macete E, Aide P, Aponte JJ, Sanz S, Mandomando I, Espasa M, Sigauque B, Dobano C, Mabunda S, DgeDge M *et al*: **Intermittent preventive treatment for malaria control administered at the time of routine vaccinations in Mozambican infants: a randomized, placebo-controlled trial**. *J Infect Dis* 2006, **194**(3):276-285.
9. Kobbe R, Kreuzberg C, Adjei S, Thompson B, Langefeld I, Thompson PA, Abruquah HH, Kreuels B, Ayim M, Busch W *et al*: **A randomized controlled trial of extended intermittent preventive antimalarial treatment in infants**. *Clin Infect Dis* 2007, **45**(1):16-25.
